# Visualization of nucleotide substitutions in the (micro)transcriptome

**DOI:** 10.1186/1471-2164-15-S4-S9

**Published:** 2014-05-20

**Authors:** Ammar Naqvi, Tiange Cui, Andrey Grigoriev

**Affiliations:** 1 Biology Dept., Center for Computational and Integrative Biology, Rutgers University, 315 Penn St, Camden, NJ 08055 USA

## Abstract

**Background:**

RNA-related applications of the next-generation sequencing (NGS) technologies require context-specific interpretations: e.g., sequence mismatches may indicate sites of RNA editing, or uneven read coverage often points to mature form of microRNA. Existing visualization tools traditionally show RNA molecules in two dimensions, with their base pairing and the resulting secondary structure. However, it is not straightforward to combine a linear NGS data display with the 2-D RNA depictions.

**Results:**

We present a novel approach for interactive representation of nucleotide substitutions and modifications in the transcribed genome. With the focus on RNA secondary structure in the context of NGS data, it provides intuitive visualization of genomic environment, sequence reads, nucleotide polymorphisms and editing events integrated with the structural and functional elements of both coding and non-coding RNA molecules. Using our approach we present and discuss examples and general trends of polymorphisms and editing in the context of the secondary structure of microRNAs. As expected, most of the substitutions comprised A to G and C to T events, consistent with typical RNA editing patterns. However, we did not observe prevalence of editing in double-stranded regions of the microRNA stem-loop. We describe novel prominent editing event candidates, observed across several small RNA libraries of *Drosophila melanogaster*.

**Conclusions:**

In contrast to the existing general tools for NGS data visualization, the power of our approach is not only in the display of read alignments and their counts, but the integration of RNA secondary structure, sequencing depth, and rates/patterns of editing or other modifications. It provides a comprehensive picture, important for large-scale studies and detailed analyses, helping to gain insight into the intricate relationships between different events in RNA biogenesis.

## Background

RNA molecules are traditionally shown in either one dimension in FASTA format or two dimensions, with the purpose of showing their base pairing and the resulting secondary structures, important for their stability and function. A number of tools for displaying RNA molecules in 2-D has been created, such as RNAViz, VARNA, RnallViewer, jViz.Rna and 4SALE [1-5], to name a few. Some of them provide not only a visual display but also tools for analysis and comparison of RNA molecules.

The advent of the next-generation sequencing (NGS) technologies has changed the research approaches in molecular biology and NGS is quickly becoming a standard. The technologies are based on determining sequences of short fragments of DNA or RNA ("reads"), assembling them *de novo *into contigs or aligning them to a reference genome sequence and finding meaningful deviation of the sequence itself or the read coverage from expected models. In RNA-related applications, these interpretations are context-specific: for instance, sequence mismatches are often interpreted as potential sites of RNA editing, or uneven read coverage is taken as an indicator of mature form of microRNA. However, it is not straightforward to combine a linear graphical display of NGS data with the 2-D RNA depictions.

Some of the tools mentioned above provide a linear view of the RNA, but none allow for clear connection with genomic features. We have developed a novel representation of RNA secondary structure that is integrated with the display of reads generated by NGS. It is similar to a linear Feynman diagram but implemented in an interactive Java applet and provides intuitive visualization of genomic environment, sequence reads, nucleotide polymorphisms and editing sites together with the structural and functional elements of the encoded RNA molecules. It is not intended to replace the 2-D depictions but, rather, to usefully supplement them with providing visual links to genomic features and experimental data, which can be overwhelming in NGS projects.

### MicroRNAs

MicroRNAs are endogenous short non-coding RNAs that are involved with regulation of messenger RNAs through either RNA degradation or translational repression. The widespread functionality of these molecules has been implicated in many different areas including development and various disease states and conditions [6]. As a result, the studies of these molecules have become essential in understanding the plasticity of the transcriptome in relation to gene expression. Emerging evidence has also surfaced suggesting that the biogenesis of these microRNAs is under precise control, which includes specific sequential cleavage, 3'-trimming and similar events [7,8].

It is important to note that the microRNA secondary structure plays a crucial role for proper processing of these molecules. For example, Drosha, an exonuclease, recognizes a transcript hairpin structure, which it then cleaves and generates a precursor microRNA. Additionally, Dicer, another protein involved with cleavage, must recognize the loop region and a specific nucleotide duplex in the microRNA stem-loop after nuclear transportation. The stem-loop length varies from microRNA to microRNA, but it includes a duplex that contains a mature microRNA and a star strand, as well as a loop region, which is removed and then subsequently degraded [7,9]. Furthermore, the strands consisting of the duplex are sometimes referred to as 5P and 3P arms or species of the microRNA stem-loop referring to their relative positions (1-based). In addition to the duplex and the loop, the stem-loop also includes small bulges across the RNA fragment. These secondary structure elements have been hypothesized to be, together with the 5' most nucleotide, the determinants of recognition-assisted partitioning and loading of the two microRNA strands into Ago1 or Ago2 complexes [10], although our report of microRNA partitioning/loading being age-dependent suggests further complexity [11].

### RNA editing

Editing is a phenomenon that is often observed in RNA and has been shown to play important roles in development, tissue specificity and RNA structure [12]. RNA editing is a molecular process in which the information content in an RNA molecule is modified through a chemical change in the base makeup. RNA editing events generally include nucleoside modifications, cytosine (C) to uracil (U) and adenosine (A) to inosine (I) deaminations, as well as un-templated nucleotide additions, deletions and insertions. These have been observed in tRNA, rRNA, mRNA and more recently in microRNA, where it has been shown that editing may be involved in target selection, degradation and stability, which greatly influence the expression and regulation of the genome [13-16].

The RNA editing is performed by the enzyme called Adar (*adenosine deaminase acting on RNA*), responsible for editing by site-specific deamination of adenosines. It specifically converts A to I. This type of editing is mostly active in the brain, but also has been implicated elsewhere, including various tissues and developmental stages [17,18]. It is also worth noting that the position of an editing event may result in truncated products, splice variants, and structural changes. All of these results may change the functionality of a particular gene and contribute to genome's plasticity making it more dynamic than originally thought. Adar has been thus far understood to function and recognize double-stranded RNA substrates [12]. Another, less prevalent, type of editing is a C to U conversion executed by ApoB/APOBEC proteins in mammals [16]. No ApoB/APOBEC homolog has been reported in *Drosophila*, although methylated C is known to spontaneously deaminate to U. In this study, we use a novel visualization approach to overlap RNA secondary structure of known *Drosophila melanogaster *microRNAs with potential editing events or other single nucleotide changes.

**Figure 1:**
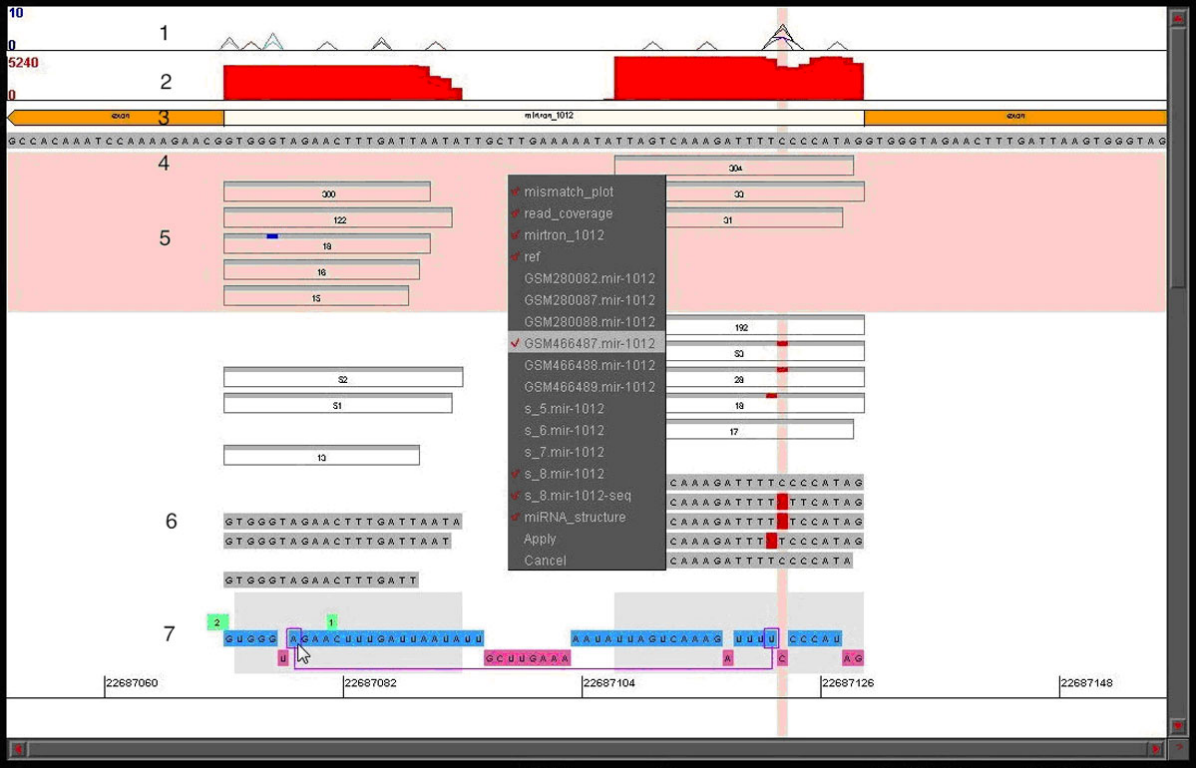
**General Display Features**. Line 1: Mismatch frequency plot. 2: Read coverage histogram. 3: Structure of the region (mirtron between two exons). 4: Reference sequence. 5: Reads & Libraries (library list in the menu in the center, selected library highlighted in pink). Identical reads are summarized as a bar with the number of reads. Colors on top of the bar show mismatches (compared to the reference genome sequence). 6: Read sequences are shown (with the similar color of mismatches). 7: Secondary structure with reference sequence from mirBase. The bottom line shows single-stranded regions in magenta. The double-stranded regions in blue are above, with base pairing encoded (if a user clicks on a position in this line, its corresponding base-pair in a stem will be connected by a magenta line in the display, as shown). Green boxes show bulge lengths in the RNA strand opposite to the bulge.

We have developed an approach to integrate the RNA secondary structure display with the results of NGS projects, using linear Feynman diagram as a model. This graphical tool is implemented in an interactive Java applet and provides intuitive visualization of genomic environment, sequence reads, nucleotide polymorphisms and editing sites together with the structural and functional elements of the encoded RNA molecules (Figure 1). Here we illustrate this visualization approach with a few examples, highlighting connections between these different sequence and structural features for microRNAs in *Drosophila melanogaster*.

## Results and discussion

### General features of visualization

As is common in the analysis of the microRNA libraries, our display shows the number of reads with the given start/end coordinates. Such numbers are often used for the determination of the borders of the 5P and 3P forms of microRNA. A group of identical sequence reads is thus summarized as a single bar with the number of reads. Further, color segments in the top line of the bar are used to highlight sequence mismatches in this group compared to the reference sequence, thus making it easier to observe specific editing patterns in a compact display. Alternatively, if necessary, individual sequences can be "spelled out" (with the similar highlighting of mismatches), as shown in Figure 1 (abundant read with a mismatch, shown under the reference sequence).

Figure 1 is an illustration of the general features of the display using *mir-1012*. Line 1 shows the mismatch frequencies or the percentage of that particular mismatch event in all reads for the specified libraries. Line 2 displays the sequencing depth of the reads mapped to the microRNA stem-loop, while line 3 shows the genomic environment (exon-intron structure for a mirtron in this case) and line 4 - the reference sequence.

Library reads are shown in line 5 (some are removed from view for compactness as described below). The list of available libraries is shown here in the menu in the center of the display and the selected library is highlighted in pink in the main display. Libraries can be de-selected to remove from view for less cluttered display. In different libraries, identical reads are summarized in a single bar with the number of counts and, when applicable, the colors on top of the bar reflect the mismatches, as compared to the reference genome.

One can also inspect actual read sequences and the secondary structure, shown in line 6 and 7, respectively. This makes the RNA secondary structure visualization intuitive and as a result we can clearly see the pre-microRNA hairpin. In this case, the secondary structure consists of single stranded and double stranded regions referring to the 5P, loop region, and 3P fragments of the stem-loop. Finally, the bottom line shows the single-stranded sections, while the double-stranded sections are above. The double stranded portion of the structure is clickable and also contains pertinent base-pairing information. In other words, if a user clicks on a position in this line, its corresponding base-pair in a stem will be connected by a magenta line in the display (as shown). Finally, the green boxes show bulge lengths in the RNA strand opposite to the bulge.

In this particular case (Figure 1), we observe that for *mir-1012 *the sequencing depth (in red) is significant in the 5P and 3P arms, indicating that both the mature and star strands are detected. We can easily extrapolate that *mir-1012 *is a mirtron and is Drosha independent, since it is located in an intronic region in the genome. More so, this visualization allows us to infer that single nucleotide changes mostly occur on the 3P arm in a bulge region. In sum, the display allows us to connect editing levels, events and locality, RNA structure, genomic regions, and NGS read counts and depth in a cohesive and meaningful manner.

### Editing

The structural complexities of microRNAs, including 5'/3' trimming, editing, and base pairing and folding, can be conveniently visualized together using our approach. These features have been shown to be very important for proper modulation and for accurate processing [7,8,19].

**Figure 2:**
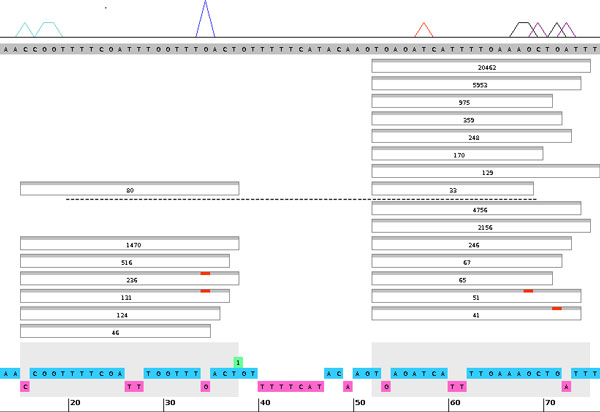
**The bantam stem-loop edited regions and NGS reads from libraries GSM280082 and GSM280087**. Dotted line separates the two libraries, with only groups containing >30 identical reads are shown. Strong editing is occurring in position 34, 71, and 68 in the stem-loop regions on both the 5P and 3P arms in the GSM280087 library (immunoprecipitated Ago1 complex). These sites are found in both single stranded (34, bulge) and double stranded regions. The top stripe plot indicates total mismatch frequencies across these and other libraries in Table 1.

For illustration, we chose *bantam *(Figure 2), a microRNA that is ubiquitously expressed and conserved with widespread associations in development and disease [20]. We identified editing events using NGS data from two different libraries, which are specific to ovaries and Ago2 loading [9], respectively. Our examples show substitutions present in both double and single stranded regions. Since Adar, implicated in most of RNA editing events, act preferentially on double-stranded regions [12], we conclude that a non Adar-mediated editing mechanism may also be involved in producing these substitutions. Our observations of nucleotide changes other than A to G also support this idea (e.g., in *bantam *and other microRNAs, data not shown). On a more global level, we were also able to identify microRNA editing candidates by analyzing several publicly available libraries (Table 1).

**Table 1:** Small RNA libraries

Library	Description
GSM280082	Ovaries
GSM280087	Ago1-IP
GSM280088	Ago2-IP
GSM466487	Total RNA
GSM466488	Ago1-IP
GSM466489	2-O Methylated
GSM811191	Nbr WT
GSM811192	Nbr -/-
GSM807162	DL-1 cells Nbr WT
GSM807167	DL-1 cells Nbr -/-

All libraries can be downloaded from NCBI's GEO database with the GSM identifiers below. Full description and references are also reported there.

For this purpose, we selected all cases where editing level (ratio of edited to total reads) exceeded a threshold of 10% with >20 reads per library. This filtering was done to avoid mismatches that result from sequencing errors. We found some 30 microRNAs that pass the filter (Table 2), with *mir-986 *and *mir-971 *being amongst the microRNAs with the highest editing level. We also observed that most of these microRNAs possess the same edits in multiple libraries furthering our confidence in these events. We also found that a C to U and A to G conversion events showed the highest level of editing (>60%) and read numbers, while other events were mostly supported by only a few reads and likely represented experimental noise. These results indicate that the canonical type and method of editing is amongst the most prevalent type and supports previous studies [12].

**Table 2:** microRNA candidates for editing

microRNA	position	reads (mismatch/total)	Level (%)	type	library
dme-mir-11	67	3192/32796	10	G>T	GSM280087
dme-mir-1001	22	152/1491	11	A>G	GSM466488
dme-mir-1010	68	11/101	11	G>T	GSM280087
dme-mir-3	54	8/78	11	A>G	GSM466488
dme-mir-312	63	3/29	11	G>T	GSM466487
dme-mir-312	65	3/29	11	T>A	GSM466487
dme-mir-970	82	381/3852	11	A>T	GSM466487
dme-mir-304	32	12/105	12	G>T	GSM280087
dme-mir-313	68	3/28	12	A>G	GSM466488
dme-mir-970	82	880/7625	12	A>T	GSM466488
dme-mir-987	35	203/1751	12	G>T	GSM280087
dme-mir-1016	23	26/214	13	T>G	GSM807167
dme-mir-2489	79	6/48	13	G>T	GSM280087
dme-mir-31b	28	15/116	13	A>T	GSM466488
dme-mir-2489	79	4/29	14	G>T	GSM466487
dme-mir-1016	23	15/95	16	T>G	GSM807162
dme-mir-317	80	2888/17496	17	G>T	GSM807167
dme-mir-308	24	1582/8795	18	G>T	GSM280088
dme-mir-4975	48	4/23	18	T>G	GSM466488
dme-mir-317	80	1946/10538	20	G>T	GSM811191
dme-mir-986	29	494/2235	23	C>T	GSM466487
dme-mir-986	29	1924/8739	23	C>T	GSM466488
dme-mir-986	29	1498/6012	25	C>T	GSM807162
dme-mir-31b	28	14/55	27	A>T	GSM466487
dme-mir-988	83	158/527	30	C>T	GSM280087
dme-mir-986	29	42/80	53	C>T	GSM280088
dme-mir-971	75	387/599	65	A>G	GSM466487
dme-mir-971	75	1675/2293	74	A>G	GSM466488
dme-mir-986	29	13057/13492	98	C>T	GSM807162
dme-mir-986	29	9276/9490	99	C>T	GSM807167
dme-mir-986	29	504/514	99	C>T	GSM280087

*mir-986*, the strongest candidate for editing, displayed 98-99% editing level in position 29 of the stem-loop. The functionality of *mir-986 *is still being elucidated, but the editing is significant due to the fact the site is 1-2 nucleotides away from the seed sequence and the event is conserved through multiple libraries. This allows us to speculate that the editing may play a major role in what mRNAs are targeted, specifically in the case of *mir-986*. The second top editing candidate microRNAs, *mir-971 *has already been reported as such in an earlier publication [19], although we did not observe editing events in their other candidates fulfilling our criteria across several libraries.

### Editing in Single- and Double-Stranded Regions

We also analyzed the frequency of these editing events in double- and single-stranded regions. We quantified all unique strong editing incidences and positions to help us understand if there was preferential editing based on RNA secondary structure of microRNA. In other words, we wanted to see if there was a significant difference in editing between bulges or double-stranded regions (the duplex) as would be expected from the properties of Adar, if it discriminated against bulges.

For single- and double-stranded regions we obtained the observed (O_ss _and O_ds_, respectively) and expected (E_ss _and E_ds_, respectively) rates of editing from publicly available libraries (Table 1) as described in Materials and Methods. The ratios of observed to expected events of O_ss_/E_ss _= 1.04 and O_ds_/E_ds _= 1.00 for single- and double-stranded editing sites, respectively, indicated that editing is essentially non-discriminatory when it comes to microRNA stem regions. We observed no editing events in the loop regions, even in those microRNAs that contained significant number of reads (>100) partially (alternative cleavage site) or entirely in loop regions, such as mir-34 (data not shown). A reason for this observation may be due to the fact that the loop regions are contiguously single stranded, which is not a canonical structure of an editing target. In contrast, single-stranded bulge regions in microRNA stems show a very slight prevalence for editing events, suggesting that Adar may tolerate bulges, if it is responsible for the editing events in microRNA.

### Differential 5P/3P abundance

As is common in the analysis of microRNA libraries, our display shows the number of counts per read with given start/end coordinates. High read numbers are often used to determine the borders of the 5P and 3P forms of microRNA. In the case of bantam, our display may reflect differential biogenesis as in some libraries abundant reads do not match the canonical end sites (Fig. 2, compare the end sites with annotated 5P and 3P forms, shown as grey shades at the bottom). This can be viewed for any set of microRNAs by inspecting start/end positions of the read and comparing it to the grey shades on the bottom of our display (annotated 5P and 3P forms).

In bantam, we can observe a high number of read counts with the end positions on the 5P arm that differ from known mirBase annotations [21,22]. We see that numerous (>100 read count) unedited reads end at position 73, while the mature microRNA is annotated as ending at position 74, thus indicating possible trimming of one nucleotide. We also observe many other highly expressed (>100 read counts) forms with different terminal sites. Additionally, the two libraries (GSM280082 and GSM280087) seem dramatically different in the assayed expression of the 5P arm (Fig. 2). While in this example we simply illustrate this difference of expression on a qualitative level, one can utilize appropriate normalization (based on RPKM, reads per kilobase per million, on read counts of endogenous siRNA, etc.) for quantitative measures to limit experimental bias or biological fluctuation.

The overall pattern of the NGS analysis of bantam suggests that Dicer cleavage can occur at multiple sites resulting in different isoforms. Notably, both of the most abundant isoforms on the 5P arm display the same substitution (editing event) in base 34. We also observe differential end positions on the 3P arm, which may hint at a 3' trimming event.

In addition to our observations and conclusions above, what makes this example more interesting is that we see in different libraries reads that correspond to only mature or both the mature and star strand. We can also see how the same microRNA is modified differently in specific libraries, which may be an effect of the cellular environment. This is a further indication that the star strand may be functional and that functionality as well as editing may be context-dependent.

## Conclusions

In this paper, we described an approach to display microRNA features and visualize them in the context of the genome environment, RNA secondary structure, nucleotide substitution and NGS. A number of visualization tools has been developed for NGS (of which the Integrative Genome Viewer [23] is perhaps the best known). What makes our approach distinct is not only the display of read alignments and their counts, but the integration of RNA secondary structure, sequencing depth, and rates/patterns of editing or other modifications. In essence it produces a more comprehensive picture, which is necessary for large-scale studies and detailed analyses. Using our approach we identified and inspected the microRNA editing candidates detected in several small RNA sequencing libraries. We observed a prevalence of canonical C to U and A to G editing patterns but found no editing bias between bulges and double-stranded regions.

Our results indicate that the modulation and modifications of microRNAs may be context-dependent or specific to experimental conditions; linking together editing, differential expression, modification, and secondary structure, helping us gain insight into the intricate relationships between these events. Finally, we can easily exploit our tool to depict other transcripts (both coding and non-coding, with and without introns) and genome/sequence features relevant for them, which makes our visualization approach both versatile and robust. The Java applet is available from the authors upon request.

## Methods

### Edit calling and filtering

Publicly available small RNA-Seq libraries were downloaded from the NCBI SRA database. Before clipping and mapping, we removed (i) the 3 adaptors from the reads, (ii) all reads shorter than 16 nucleotides and (iii) those with one or more "N" nucleotides present. The remaining reads were then mapped against the stem-loop sequences obtained from mirBase [22] using Bowtie ver. 1.0 (-v 1, -a options) [24]. Reads that mapped to the microRNA stem-loops with one mismatch were then mapped against the whole *Drosophila *genome (dm3) using Bowtie. In order to filter out potential noise and artifacts, all mismatched reads that also successfully aligned elsewhere in the genome with at most one mismatch were removed. These steps were performed for each of the ten libraries utilized in this study. We then quantified observed mismatches for every position in all known microRNAs in *Drosophila*.

To limit the experimental artifacts, we used specific thresholds for read count (>20) and editing rate (>10%), multiple library and sequence mappings (i.e. different sequences from the same library having the same position edited and different sequences from different libraries having the same position edited), and other similar measures. While we cannot guarantee that we removed all sequencing artifacts, they are significantly reduced with such filtering.

### Evaluating editing events in structural context

MicroRNA secondary structure annotation was taken from mirBase [22]. For our random model, with editing independent of the secondary structure, we determined the expected number of edits occurring in bulge (E_ss _) and double-stranded regions (E_ds _) as follows:

$${\text{E}}_{\text{ds}}={\text{S*N}}_{\text{ds}}/\left({\text{N}}_{\text{ds}}+{\text{N}}_{\text{ss}}\right)$$

$${\text{E}}_{\text{ss}}={\text{S*N}}_{\text{ss}}/\left({\text{N}}_{\text{ds}}+{\text{N}}_{\text{ss}}\right)$$

where S is the total number of edits observed and N_ds _and N_ss _are the total numbers of nucleotides in double- and single-stranded regions, respectively. We then obtained ratios of observed vs expected events for these region types and did not detect any significant difference, with a slight prevalence of the observed single-stranded edits.

## Competing interests

The authors declare that they have no competing interests.

## Authors' contributions

TC wrote the visualization program and scripts for data preparation, AN performed the data analysis and prepared the data for visualization, AG conceived and directed the study, wrote the visualization program. AN and AG wrote the manuscript.

## Declarations

The publication costs for this article were funded by Rutgers University.

This article has been published as part of *BMC Genomics *Volume 15 Supplement 4, 2014: SNP-SIG 2013: Identification and annotation of genetic variants in the context of structure, function, and disease. The full contents of the supplement are available online at http://www.biomedcentral.com/bmcgenomics/supplements/15/S4
